# Supplementary material for: Learning curve and surgical outcome of robotic assisted colorectal surgery with ERAS program
Source: Sci Rep. 2022 Nov 29;12:20566. doi: 10.1038/s41598-022-24665-w (PMC9709162; doi:10.1038/s41598-022-24665-w)
Supplement: Supplementary file 1 — Supplementary Information 1. [file 41598_2022_24665_MOESM1_ESM.zip › LSC Raw data-Ñ[▒K/056/OP N_ñw╝╨░O▒Kñσ.pdf]

W55 / W55

臺中榮民總醫院
手術室護理記錄

[redacted]

基本資料

- 室別： 二醫手術室-31室，手術科別： CRS，手術類別： 常規，日期： 2019/12/12
- 進入時間： 1145 ； 麻醉開始時間： 1148 ； Time Out時間： 1250 ； 劃刀時間： 1251 ； 手術結束時間： 1700 送出時間： 1715 ；
- 麻醉方式： GE
  診斷：Malignant tumor of rectum SP CCRT
  手術名稱：1.Low anterior resection with colo-anal anastomosis2.Ileostomy3.Laparoscopy4.
  手術局部用藥：無
  術前預防性抗生素：
  - 抗生素名稱一： Veterin 1GM IVD/由麻醉護理執行
  - 時間： 1220
  - 上次簽章: 彭馨慧

  ，追加預防性抗生素：
- 手術醫師： 蔣鋒帆
- 麻醉醫師： VS 賴慧卿
-
  - 刷手護士A ： 林昱君，時間： 1145-1200，接替刷手一： 謝昀安，時間： 1200-1230，接替刷手二： 林昱君，時間： 1230-1300
  - 流動護士A ： 彭馨慧，時間： 1145-1300，接替流動一： 林昱君，時間： 1300，接替流動二： 彭馨慧，時間： 1530
- 更多接替及流動護士： 接替刷手三： 顏育銹，時間： 1300

皮膚狀況

A-V Shunt： 無
術前： 完整
記錄者： 上次簽章: 彭馨慧
術後： 完整
記錄者： 彭馨慧

手術準備

手術臥式 單· ·手術臥式

-
  - 手術臥式： 截石臥式
  - 使用附件： 溫毯 38 ℃，頭圈，腳架
  - 皮膚準備： 10%Aq Betadine，Hibiscrub，其它： 2%chlorhexcidine
  - 電燒： 使用電燒: 電極片位置： 其它： 迴路電極墊，記錄者： 上次簽章: 彭馨慧

止血帶T： 無

植入物

- 植入物名稱/號碼/數量/部位：12mm clip applier /colon
  endo GIA 60/4.1 /colon
  EEA 33mm /colon
  Surgiwrap 100*130mm/pelvic

, 記錄者： 上次簽章: 林昱君

標本送檢

- 細菌培養 無
- 細胞學 無
- 冰凍切片 無
- 病理標本
  組織名稱一：rectum
  ,
  組織名稱二：IMA root

,
組織名稱三： distal cutend

- 截肢 無

標本交付 標本： 3 袋 ， 標本： 0 盒 ， 標本： 0 瓶 ， 細胞學： 0 管 ， Culture Tube： 0 管 ， 被交付醫師： R 陳璿夫 ，

- 記錄者： 彭馨慧
- 時間： 1850

傷口術後數料

傷口數料： 傷口數料： 無

- 請於圖上標示部位：

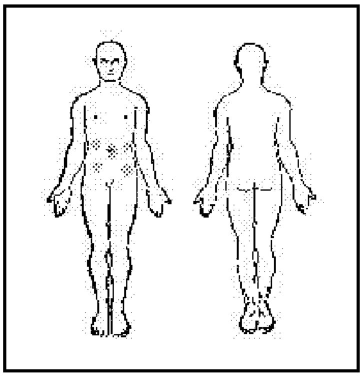

- 傷口數料： 腹部3個傷口menpore覆蓋

導管

導管	天數	導管資訊	插入日期	插入時間	更換日期	拔除日期	拔除時間	拔除原因	預計拔除/更換	預計拔除/更換日期
Rod,Stoma Supporting (1)	1	部位:ileostomy;	2019/12/12	1600						
Sump NG Tube(1)	1	號碼:16 Fr.; 部位:胃;	2019/12/12	1218						
Double-J (1)	1	號碼:6Fr*26cm ; 部位:輸尿管; 註記:left;	2019/12/12	1650						
Penrose Drain(1)	1	號碼:12 mm.; 部位:骨盆腔;	2019/12/12	1500						
Foley(1)	1	號碼:18 Fr.; 部位:尿道;	2019/12/12	1215						
A-line(1)	1	號碼:20 號; 部位:右手;	2019/12/12	1210						

用物計數

- 手術中出血量： 少量 c.c.
- 手術中尿量： 250 c.c.
-
  - 其它： 無

用物計數： 單一切口

- 1.器械： 正確 ，2.縫針： 正確 ，3.刀片： 正確 ，4.紗布： 正確 ，5.腹部墊： 未使用 ，6.紗球： 未使用 ，7.PEANUTS ： 未使用 ，8.紗條： 未使用 ，9.綵帶： 未使用 ，10.針頭： 未使用 ，11.Vessel loop： 未使用 ，12.Boots： 未使用 ，13.Jelco： 未使用 ，14.棉條： 未使用 ，
  - 第一次：
    - 刷手護士 ： 顏育琇
    - 流動護士： 上次簽章: 林昱君
    - 時間： 1500

  ,
  - 第二次：
    - 刷手護士 ： 顏育琇
    - 流動護士： 上次簽章: 林昱君
    - 時間： 1520

  ,
  - 第三次：

  - 刷手護士 ： 顏育琇
  - 流動護士： 彭馨慧
  - 時間： 1540

備註

備註： NIL

- 記錄者： 彭馨慧
- 時間： 1700

填寫護理人員 彭馨慧

正式摘要

W55 / W55

doctorRank:B
